# Tendon lengthening and fascia release for healing and preventing diabetic foot ulcers: a systematic review and meta-analysis

**DOI:** 10.1186/1757-1146-8-S2-O10

**Published:** 2015-09-22

**Authors:** Dallimore Sarah, Michelle R Kaminski

**Affiliations:** 1 Department of Podiatry, Eastern Health, Box Hill, Victoria, 3128, Australia

**Keywords:** Achilles tendon lengthening, diabetic foot ulcer, gastrocnemius recession, plantar fascia release

## Background

Diabetic foot ulcers have a devastating impact on an individual's health-related quality of life and functional status. Additionally, diabetic foot ulcers impose a significant economic burden on our health care systems as a result of complications such as infection, hospitalisation and amputation. The current gold standard treatment is total contact casting. However, the rate of ulcer recurrence is high indicating the need for more effective long-term treatment options. The aim of this study was to evaluate all literature investigating the effectiveness of Achilles tendon lengthening, gastrocnemius recession and selective plantar fascia release in healing and preventing diabetic foot ulcers. The primary outcome measures were time to healing of the ulcer, rate of ulcers healed, rate of ulcer recurrence and rate of transfer ulcers (i.e. new ulcer). The secondary outcome measures were complications and adverse events.

## Process

Searches were conducted in MEDLINE, CINAHL, AMED, EMBASE and The Cochrane Library from the earliest available date to November 2014. Methodological quality of included studies was assessed using the Downs and Black checklist. Where possible, data were analysed using random effects meta-analyses.

## Findings

Eleven studies were included in the review; two randomised-controlled trials, one prospective cohort study, three prospective case series, two retrospective cohort studies and three retrospective case series. Time to healing of diabetic foot ulcers was not significantly different between Achilles tendon lengthening or gastrocnemius recession and total contact casting (MD = 8.22 days, 95% CI -18.99 to 35.43, P = 0.55). Overall, 91% of ulcers healed, 11% experienced ulcer recurrence, and 8% developed a transfer ulcer following Achilles tendon lengthening, gastrocnemius recession or selective plantar fascia release.

## Conclusions

Achilles tendon lengthening, gastrocnemius recession and selective plantar fascia release appear to be effective surgical treatments in healing diabetic foot ulcers. In addition, the rate of ulcer recurrence has been found to be lower than the gold standard treatment of total contact casting. Therefore, these surgical procedures may provide a viable adjunctive treatment option for the management and prevention of diabetic foot ulcers. Further rigorous randomised-controlled trials with longer follow-up are required to determine the long-term effectiveness and safety of these procedures.

